# Experiences From a Web- and App-Based Workplace Health Promotion Intervention Among Employees in the Social and Health Care Sector Based on Use-Data and Qualitative Interviews

**DOI:** 10.2196/jmir.7278

**Published:** 2017-10-19

**Authors:** Nina Charlotte Balk-Møller, Thomas Meinert Larsen, Lotte Holm

**Affiliations:** ^1^ Department of Nutrition, Exercise and Sports University of Copenhagen Frederiksberg C Denmark; ^2^ Section for Consumption, Bioethics and Governance Department of Food and Resource Economics University of Copenhagen Frederiksberg C Denmark

**Keywords:** eHealth, health promotion, workplace, smartphone, weight reduction programs, Internet, qualitative research

## Abstract

**Background:**

An increasing number of Web- and app-based tools for health promotion are being developed at the moment. The ambition is generally to reach out to a larger part of the population and to help users improve their lifestyle and develop healthier habits, and thereby improve their health status. However, the positive effects are generally modest. To understand why the effects are modest, further investigation into the participants’ experiences and the social aspects of using Web- and app-based health promotion tools is needed.

**Objective:**

The objectives of this study were to investigate the motivation behind taking part in and using a Web- and app-based health promotion tool (SoSu-life) at the workplace and to explore the participants’ experiences with using the tool.

**Methods:**

Qualitative interviews with 26 participants who participated in a 38-week randomized controlled trial of a workplace Web- and app-based tool for health promotion were conducted. Data were supplemented with tracking the frequency of use. The basic features of the tool investigated in the trial were self-reporting of diet and exercise, personalized feedback, suggestions for activities and programs, practical tips and tricks, and a series of social features designed to support and build interactions among the participants at the workplace.

**Results:**

The respondents reported typically one of the two reasons for signing up to participate in the study: either a personal wish to attain some health benefits or the more social reason that participants did not want to miss out on the social interaction with colleagues. Peer pressure from colleagues had made some participants to sign up even though they did not believe they had an unhealthy behavior. Of the total of 355 participants in the intervention group, 203 (57.2%) left the intervention before it ended. Of the remaining participants, most did not use the tool after the competition at the end of the initial 16-week period. The actual number of active users of the tool throughout the whole intervention period was low; however, the participants reported that lifestyle habits became a topic of conversation.

**Conclusions:**

A tool that addresses group interactions at workplaces appears to initiate peer pressure, which helped recruitment for participation. However, active participation was low. A social change was indicated, allowing for more interaction among colleagues around healthy lifestyle issues. Future and more long-term studies are needed to determine whether such social changes could lead to sustained improvements of health.

## Introduction

### Background

The number of Web- and app-based tools for potentially inexpensive health promotion that are being developed at the moment is increasing. Mobile phone apps and websites are being designed to help users keep track of their behavior, develop healthier habits, and improve their lifestyle [1]. At the same time, the workplace is gradually becoming more used in health promotion because many people spend a lot of their waking hours at work, and the World Health Organization has declared the workplace a prioritized arena for health promotion [2]. Lifestyle interventions are needed to combat the increasing prevalence of obesity and diseases related to unhealthy lifestyle. In Denmark, this is especially the case in people with low education attainment.

Social and health care workers in Denmark have comparatively less education and lower health status than the average population [3]. They generally smoke more and are more overweight [4]. Furthermore, they have a higher level of absence from work and a higher risk of leaving the workforce early because of sickness.

Scientific research on apps and websites for weight loss is still evolving, and the results from these studies show that the effects are generally modest, of a limited duration, or inconclusive. In addition, two recent systematic reviews with meta-analysis on the use of mobile devices or apps for weight loss found that the use of these tools induced weight loss [5] but did not have an effect on physical activity [6], and another recent study found promising results using a Web-based app for promoting healthy lifestyles [7]. Studies evaluating Web-based weight loss programs [8,9] and the use of text messaging (short message service, SMS) for weight loss [10] found positive results. Furthermore, in a review, eHealth tools for physical activity and dietary behavioral change were found to have the potential for improving these issues [11]. All these features are included in the SoSu-life tool (see Methods section).

A systematic review on workplace health promotion for healthy eating and physical activity found the evidence for positive effects of the interventions to be limited to modest [12], and another review targeted to increase physical activity found that the interventions can be efficacious, but the overall results were inconclusive [13]. However, a recent meta-analysis found that workplace health promotion interventions resulted in improvements in self-perceived health, decreased absence due to illness, and increased productivity [14].

Workplace interventions in social and health care workers have been addressed in a few studies from Norway, with clinical data as well as subjective measures as outcomes. One study evaluated health promotion of physical exercise at the worksite in nursing homes and unexpectedly found an increase in sickness absence in both intervention and control groups during the study period [15]. Another study including physical activity found no improvements in health-related quality of life and no difference in sickness absence between the two groups [16]. Therefore, the results regarding the effect of workplace interventions in social and health care workers are indecisive.

Although the clinical effects of health interventions have been investigated, insight into *why* interventions work at a practical and social level is less frequently examined. There is very little information about the participants’ subjective experiences, their use of the tools, and the social settings of the interventions in the available literature. Examining these parameters might give some insight into why the effects found in these intervention studies are generally modest [17]. In this paper, we explore practical and social experiences of using a Web- and app-based tool for health promotion.

In 2012, a Web- and app-based tool for health promotion at the workplace was developed (called the SoSu-life tool), targeting social and health care workers in Denmark to help them to make lifestyle changes. The health promotion tool included behavior change techniques (BCTs) at both the individual and the social level. The tool’s main feature is the individual feedback system, which operates on the individual level in the BCT taxonomy [18,19]. The tool also entails social features such as team competition operating on a social level. All features were designed to encourage health-related changes for the individual participant. It was designed to work both at nursing homes, where colleagues work side by side, and at home care units, where colleagues work individually in elderly citizens’ homes and only meet with workmates for lunch breaks or short daily meetings. In the SoSu-life study, the tool was evaluated in a 38-week randomized controlled trial. The results from this study were modest [20].

### The Aim of This Study

In this study, we examine the participants’ experiences with the tool both by analyzing use-data and by conducting interviews with the participants. We examine how the tool was used during the intervention period and which features were most popular in a simple descriptive manner. In the interviews, we wanted to investigate what kind of motivation the participants had for using the tool and which changes in lifestyle behavior occurred at the individual level as well as in the group interaction.

## Methods

### Setting: The SoSu-Life Intervention Study

**Figure 1:**
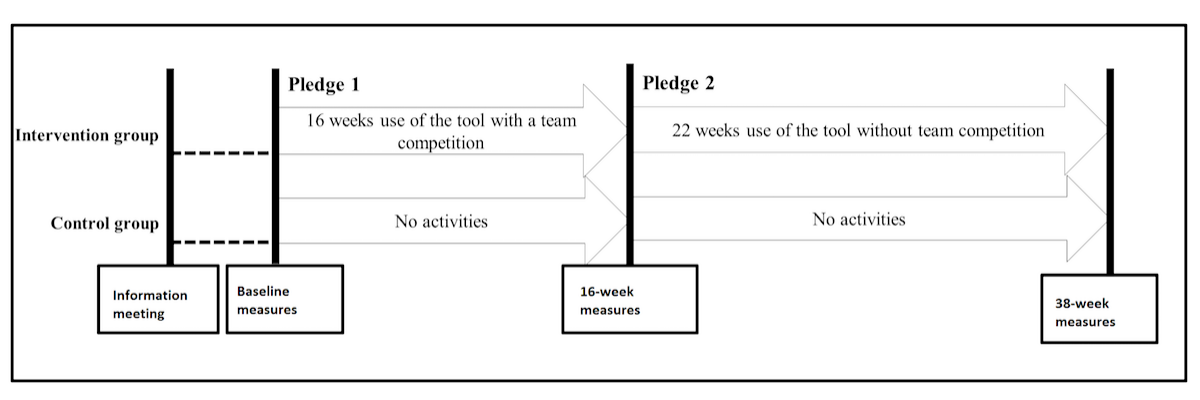
Overview of the SoSu-life study.

In 2012, a 38-week cluster randomized controlled intervention study (NCT02438059) among 556 employees in the social welfare and health care sector (SoSu’s) in Denmark was carried out (overview of the study presented in Figure 1). In total, 6 municipalities agreed to participate, and in each municipality, between 2 and 5 nursing homes or home care units signed up, covering a total of approximately 1203 potential study participants. A total of 12 units were randomized to the intervention group and 8 to the control group with no treatment. The intervention group went through an initial 16-week period with team competition, and a 22-week follow-up period without team competition. The control group had no activities, but both groups went through a health examination at weeks 0, 16, and 38. Both groups were aware of the overall study design and also that the aim of the intervention was to promote individual health of the participants.

**Qualitative Data Collection**

The qualitative data were collected using personal interviews (n=24) and focus group interviews (2 groups with 7 in each; n=14), among both active users of the SoSu-life tool and nonusers of the tool in the intervention group. Of the health care units in the intervention group, 4 out of 12 were represented in the qualitative data. The participants for the interviews were recruited by phone and selected according to their earned points, which can be seen as an expression of how much the user uses the tool; participants with both low and high number of points were selected for the personal interviews to ensure that both positive and negative experiences were collected. Participants with low, middle, and high number of points were recruited for the focus group interviews. The first round of interviews were conducted approximately 8 weeks after baseline health examination and the second round of interviews approximately 8 weeks after the health examination held after 16 weeks.

An interview guide was developed, making sure the interviews covered all aspects of the intervention. The participants were encouraged to talk freely about their experiences with participating in the project and using the tool. They were asked to describe in detail how they had been introduced to the project, their experience with the health examinations, how they used the tool, which features they found useful, and which features they were particularly critical of. They were prompted to reflect on the changes they had experienced individually and at the workplace during the intervention period.

**Figure 2:**
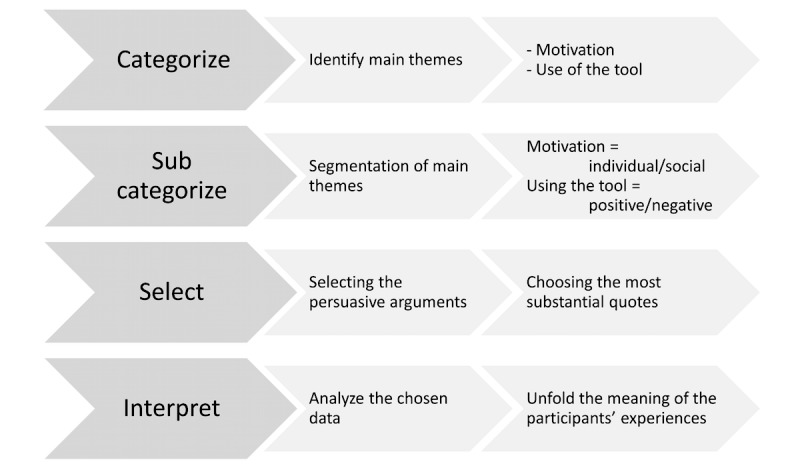
Steps in the analysis coding process.

The interviews were transcribed and coded according to the standard qualitative analysis procedure to themes related to motivation, and use of and experiences with the different features in the tool. In the second round of coding, special attention was paid to whether the participants’ experiences were positive or negative and whether the motivation for joining the study was for individual reasons or for social reasons. Then the relevant arguments were considered and substantial quotes were chosen. Finally, the interpretation of the chosen data was done (Figure 2).

### Use-Data Collection

In this paper, we further present a simple descriptive analysis of data about the participants’ use of the tool. The user statistics were collected centrally from the distributor of the SoSu-life tool during the intervention period. In this analysis, data consist of the pledges among the participants, number of collected points, number of days diet and exercise were registered, and number of accepted weekly assignments and sent *colleague challenges* (all described in the following section). The user data were extracted from the database at the end of the intervention at week 38.

### Description of the SoSu-Life Tool

The SoSu-life tool’s basic features are self-reporting of diet and exercise, personalized feedback, suggestions for activities and programs, practical tips and tricks, and a series of social features, including weekly assignments and colleague challenges designed to support and build interactions at the workplace. The SoSu-life tool aims at mobilizing whole groups of colleagues not only to encourage each other in achieving personal goals but also to have all group members work on identical small weekly assignments. Points were assigned to all individual and group activities and were collected by both individuals and groups. Individual activities gave points not only to the individual but also to the group, as part of the group competition. In this way, each individual’s use of the digital tool benefited the whole group.

Each participant was given a 10- to 15-min introduction to the website and the app by a member from the project team and was provided a pamphlet with information on content and functionalities of the tool. When the user signed up to use the tool during the introduction, she or he chose 1 pledge out of 7 to focus on the following: lose weight, eat healthier, improve physical fitness, improve physical strength, quit smoking, decrease the number of cigarettes, or maintain a healthy lifestyle. The program itself would indicate a recommended pledge based on individual information from the health examination. The choice of pledge influenced the features and feedback provided by the SoSu-life program, such as the frequency and content of emails and SMS texts sent to the participant. The messages contained information about specific health issues related to the pledge, general tips, and tricks on health and well-being.

The program had different functional tools to help the user succeed with the pledges. The self-reporting of diet and exercise functioned as a weight loss tool based on a unit system. All foods were assigned a number of units based on the portion size, calories, and macronutrient composition. Daily energy level was calculated based on the user’s height and weight, and the number of units that should be consumed per day for losing weight was suggested. The user registered his or her food intake and daily exercise, and the program gave feedback on the energy balance of the day, a green code indicating a proper energy balance and a red code for excessive energy intake. Exercise was registered as bonus units so that the user could compare the number of units earned from food consumption with the number of units earned from exercise (the bonus units). The same system was used for those participants wishing to focus on exercise alone, and feedback was given in the form of the number of bonus units earned. Additionally, the website provided access to a number of suggested video-supported exercise programs to increase fitness level or improve strength. Smokers wishing to either change their smoking habits or quit smoking were advised to begin by registering their habitual use of cigarettes, the time the cigarettes were smoked, and the mood they were in when they were smoking the cigarettes.

The social features included shifting weekly assignments for the whole group of participating colleagues. Such weekly assignments could be *drink at least one liter of water every day all week* or *remember to say Good Morning to your colleagues every morning all week*. The tool also included *colleague challenges*, which were sent from colleague to colleague and were determined by the participants’ individual pledge. Challenges might be *do not eat sugar for three days* or *bring some fruit for us to eat together tomorrow during the afternoon break*.

**Figure 3:**
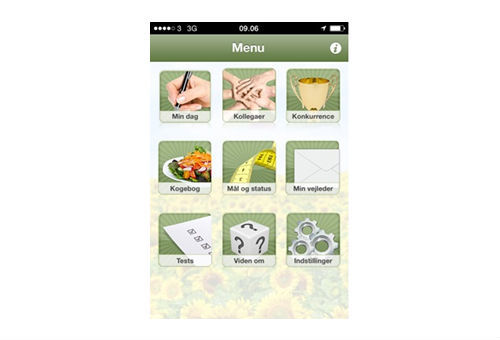
SoSu-life app main menu.

**Figure 4:**
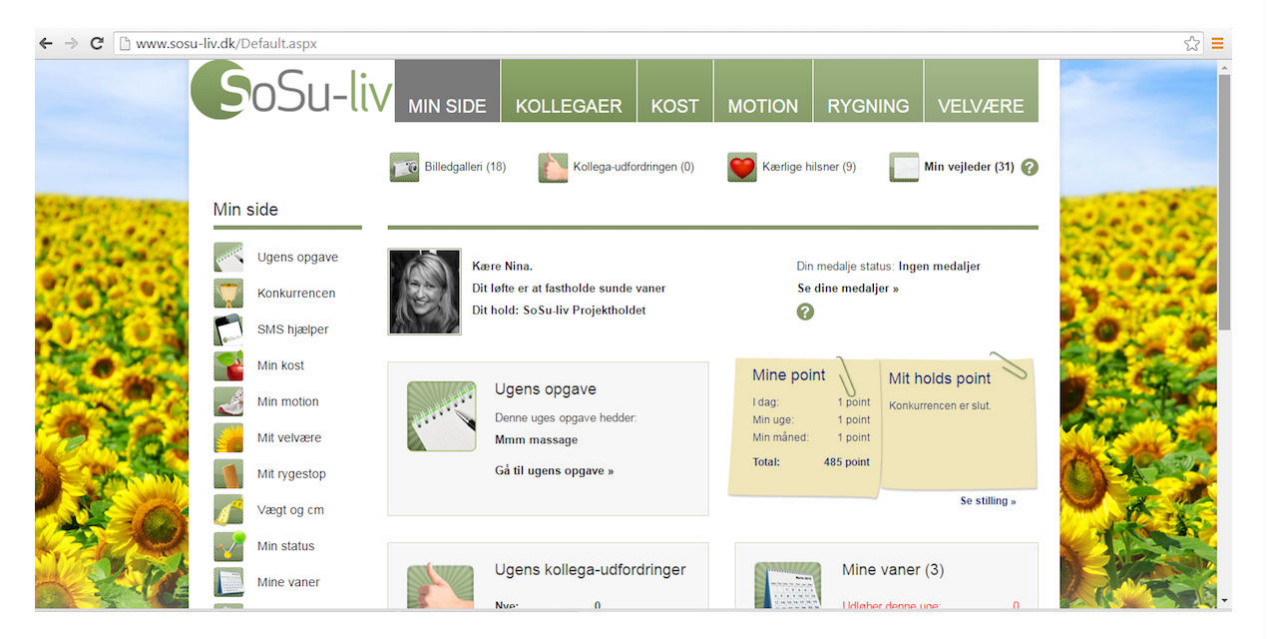
SoSu-life website frontpage.

All features could be accessed from both the app and website (Figures 3 and 4)

The SoSu-life tool used a point system where all activities performed using the tool gave points to the individual user. The point system provided the highest reward for taking part in social activities. Performing the weekly challenges and sending and carrying out *colleague challenges* were rewarded with more points than registering diet or exercise or taking tests or quizzes. During the first 16 weeks, each of the participating nursing homes or home care units constituted a team of participants, and each of the user’s individual points were added to the team’s total points. A ticket was generated for every point collected by the team, and this was put in a lottery. Each month, the teams had a chance to win a prize by a simple lottery. The more points the team had, the bigger the chances were of winning. The prizes could be a shopping bag for each team member, a Zumba class for the team, or a visit from a bartender who served fruit smoothies during lunch hours. The team with the most points after 16 weeks also won a prize. Points were still collected in the second (22-week) intervention period, but no prizes were provided. All prizes were provided by the main sponsor of the project. The social features were designed to create a supporting atmosphere to help generate behavior change for the individual participant.

## Results

### Registered Use of the SoSu-Life Tool

**Figure 5:**
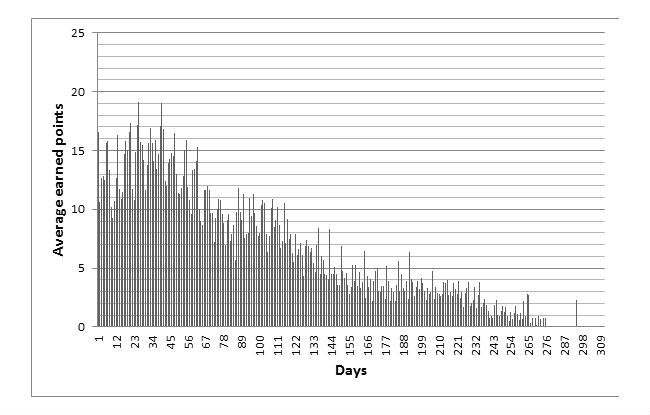
Average points earned per day.

The use-data gave insight into the engagement with the SoSu-life tool and which features were most popular. Points are an indicator on how much engagement the participants had with the tool, as they earned points according to the activity they performed or the feature they used in the tool. A general overview of the average points earned per day (Figure 5) shows that it was more popular to use the tool during the first 16 weeks where the team competition took place. But the gradual decline of use began around 40 days, indicating an even earlier drop of interest in the tool.

**Figure 6:**
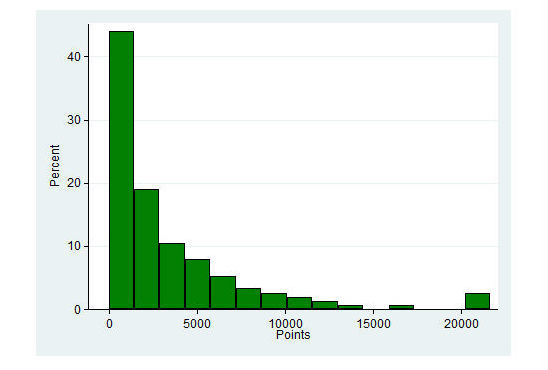
Distribution of total amount of points earned during the 38-week intervention period (n=152).

At the baseline health examination, all participants earned between 1 and 40 points when they were introduced to the tool by the SoSu-life project workers. Figure 6 shows that approximately two-thirds of the participants only made a few extra points during the rest of the intervention period, meaning that they did not really use the tool actively after the introduction.

**The Diet and Exercise Modules**

Registering physical activity worked in the same way as registering diet, with feedback provided in the form of bonus units, depending on the time and type of exercise performed. It required less time and effort from the user to register one or two types of exercises compared with a full day’s diet, which made it easier for the participants to use. Furthermore, the exercise feature appealed to both participants with pledges on losing weight and those choosing to improve physical fitness and strength.

**Figure 7:**
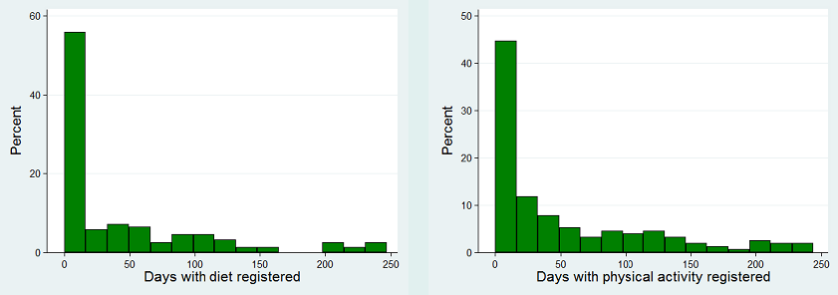
Distribution of days with respective registered diet and exercise during the 38 weeks (n=152).

It appears that the number of days registering exercise was slightly higher than for those registering diet (Figure 7). More participants tried the diet registration (about 58%) compared with those who tried the exercise module (about 44%) because the diet module was a part of the standard introduction to the tool. However, most of the participants stopped using both parts of the tool after a while.

### The Social Features in the Tool

**Figure 8:**
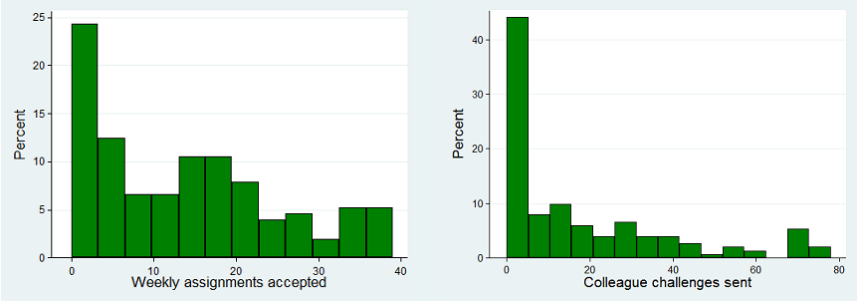
Distribution of number of days for weekly assignments accepted and “colleague challenges” sent out (n=152).

The SoSu-life tool had social features that were meant to improve social interactions and group dynamics at the workplace. These were most popular during the first 16 weeks of the intervention period. These social features seemed to be used slightly more (Figure 8) than diet and activity registration (Figure 7).

### Findings From the Interviews

#### Motivation

From the interviews conducted, we found that the participants were motivated by different aspects of the intervention. Some were motivated by the prospect of getting help to lose weight, eat more healthily, or exercise more, including the feedback on dietary habits and health behavior. Others were motivated by the anthropometric and clinical examination, where getting the physiological data on their bodily measures worked as an incentive to change their lifestyle. None of the respondents mentioned the chance of winning prizes as a reason for signing up when they were asked an open question on why they signed up for participation, but the social part of being a team in the competition was a reason for continually using the tool during the first 16 weeks. When the project was presented to the participants, the social interactions and community elements were emphasized as important factors, and the participants and daily leaders of the workplaces encouraged each other to take part in the project. This meant that participants with no specific individual motivation for making lifestyle changes or no obvious unhealthy lifestyle also signed up for participation in the intervention.

#### Experiences With the Diet and Exercise Modules

In the interviews, some of the participants expressed that they found the tool too technically difficult to use or too time-consuming, whereas others did not think the weight loss tool was useful for them.

The participants who used the self-monitoring tool to keep track of their food intake and exercise level said that they had a starting period where they had to learn how to use the features. Only a small number of participants really changed their eating habits using the SoSu-life weight loss tool.

As explained here, by a very active user:

Yes, I actually type almost straight after [I have eaten] and sometimes I will make the food ready, and I will type before I eat. Someone tells me, you must not get stressed by it, because people say you tend to sit and type before you eat. (...) My partner says, by now, you must be able to remember what you eat and then type afterwards. Now, concentrate on eating your food.

Here, another participant is talking about how the unit system is constantly giving her feedback on her diet habits:

But really, it ensures an increased focus all the time because one sees the damn units every time one types it in. And then I just need to learn to weigh the food before I eat it.

Using the unit system has transformed these participants’ relationship to food. The app had become an integrated element in their relationship to food and eating, as it was telling them how much more they could eat or how much more they should exercise. The typing of food intake together with using the field system thus gave those users a more instrumental relationship to food.

Others registering diet for a short period gained new knowledge about the food they ate, which could help them attain a balanced diet. For example, the unit system was still present in a participant’s consciousness when choosing whether to eat a piece of cake:

No, I don’t know if I want to use units for this—it’s a little funny. Because, this means, that now I’m more conscious about what I consume, and also how much it costs in units.

Others viewed the feedback with ambivalence. When they registered the actual food they ate, the tool highlighted them in a red color when they ate too much, which gave them a bad conscience and made them want to drop using the tool.

But, I just get cravings to eat a cake when I think it gets damn annoying with all this typing. And then I just eat a cake and type it in, and then that day is just wasted.

This participant was critical about her colleagues being so focused on food that she felt like deliberately eating unhealthy food as some kind of protest. Furthermore, when the feedback from the unit system was negative, she felt the day was wasted. Instead of regulating her calorie intake, she became really frustrated and acted with resistance toward healthy food and gave up using the tool completely.

On the basis of the findings above, we interpret that the individual feedback mechanisms in the tool are of great importance; however, they work in very different ways for the participants. Some participants learned how to eat a more balanced diet because of the feedback the tool gave them. However, others simply dropped using the technology when the feedback was negative to their habits. Only those participants who were motivated by the control system of registration and feedback were able to overcome the practical and technological challenges and became comfortable and at ease with using the daily registration system.

#### Experiences With the Social Features of the Tool

Both weekly assignments and colleague challenges were designed to encourage the whole workplace to engage in the project and to support each other. These features were designed to create a *we-are-in-this-together* spirit. At some nursing homes, the SoSu-life project was embraced with enthusiasm. In those places, the participants spoke about a change in how the colleagues engaged with each other and about the norms related to the participants’ personal health. At these locations, the norms relating to how the colleagues addressed each other regarding health habits seemed to change, which is illustrated in the following quotes from the respondents. It became legitimate to approach a participating colleague and ask how her weight loss was going or to ask a smoking colleague how her quitting smoking project was coming along.

In a group interview approximately 6 weeks into the intervention period, participants discussed how they now talked more about their habits regarding smoking and food. One participant said:

But Susanne asked me the other day—well, have you been blowing on your cigarette [refers to a newly purchased electronic cigarette]. She would not have asked me that, if we weren’t participating in this.

Another participant explained:

That we have had this dialogue back-and-forth with each other and that it has been completely legitimate to stop each other in the halls and say “say…how much weight have you lost.” We had not done this before, even if there was someone who had lost weight and it was visible.

Furthermore, new social bonds were formed as colleagues began interacting with other participants, sending messages and talking to colleagues they did not talk to before the project, whereas others formed small groups supporting each other in keeping the healthy habits.

The SoSu-life tool gave the participants a reference within which to engage in each other: to talk about food, talk about weight loss, talk about smoking, and a common reference regarding healthy habits.

Some of the active users of the SoSu-life tool mentioned that they had an agenda of replacing unhealthy elements in their workplace with healthier ones, such as having more fruit instead of cake available at the workplace.

With the introduction of SoSu-life tool, there seemed to be a change in the social interactions at the workplace in terms of what was legitimate to talk with other colleagues about. They now discussed weight loss and smoking cessation with less hesitation—issues that were previously considered private matters. However, the change was not only positive. For some participants, the constant focus on health and food was followed by an aversion to being healthy. Thus, the social features, with the chatting and social interaction tool, also had a tiring effect on some of the participants.

A participant expressed her opinion about the project after the first 16 weeks:

It was not like this in the beginning, but now...I think the others are doing really well with the tool, and I don’t know why this [demotivated and exhaustion regarding the project] is happening to me. I just think I have too much to deal with at the moment. I’m really tired of it. I’m really tired of all the typing and I’m tired of...because it doesn’t matter where ever I turn people are talking about some sort of food. And this is what I’m tired of because this is exactly what I wanted to stop, having to focus on food.

## Discussion

### Principal Findings

The study found that only very few participants used the SoSu-life tool throughout the project period. Especially, the individual features of the tool were rarely used, whereas the social features were somewhat more popular. The overall clinical health benefits in this project [20], as in other similar projects, were minor, but the SoSu-life project seems to have initiated some potentially positive changes in the social interaction among colleagues, although the increased focus on healthy eating turned out to be demotivating for some participants. Furthermore, the social features of the tool meant that more social and health care workers signed up to participate in the project, which was positive. On the other hand, the limited use of the individual features of the tool suggests that although participation was prompted by the social features, these were not sufficient to motivate engagement with the individual features of the tool and with individual lifestyle change.

### Interpretation

In the literature, incentives were described as important for signing up to participate in workplace health promotion [21]; however, in this study, none of the respondents reported the prizes in the competition as a reason for signing up, but merely the social aspect of being part of a team.

It should also be taken into consideration that the SoSu-life project was the first time some of the social and health care workers tried to use a smartphone. Clearly, participants with no specific motivation or limited technical skills had strong odds against them being active users of the SoSu-life tool. They would probably have benefited from a more thorough introduction to the functionalities of both the smartphone and the website.

In both Web- and app-based health promotion and workplace health promotion, a general problem is the high level of dropout and attrition in the use of the tools [21,22]. This is also the case in this study. In the literature, high dropout rate is considered to be natural and typical, and the fact that participants stop using the digital interventions has even been called one of the fundamental characteristics and methodological challenges in the evaluation of eHealth interventions [22]. A scientific review investigating participation in worksite health promotion programs found that only half of employees are usually reached in workplace-based intervention, and another review found that typically around one-third of participants left worksite health promotion programs early [21,23].

Strategies designed to help participants change their behavior with an aim to adopt a healthy lifestyle may be implicit or explicit. The BCT taxonomy categorizes the specific strategies that are used in interventions to promote behavior change, ranging from techniques that work on the individual to techniques that work on the social level [18,19]. Currently, and most commonly, health interventions are described as using techniques focusing at the individual level. This category is differentiated with several subcategories within the BCT framework. Interventions using techniques on the social level are less often reported. Furthermore, the social level category is only described with the broad term “planning social support or social change” [18]. The SoSu-life tool includes several mechanisms that operate on the social level. The results of this study indicate that the BCT framework should be developed with more refined categories of social techniques. It is relevant to distinguish between mechanisms that ensure that individual activity benefits the social group, mechanisms that initiate joint activities, mechanisms that create team spirit, and mechanisms that initiate team-based competition. Our findings give a more nuanced view on how Web- and app-based health promotion tools work. They suggest that it might be the social interactions and conversations among colleagues at the workplace initiated by the intervention, rather than individual use of the tool, that create actual behavioral changes, thus influencing whether the whole intervention as such is beneficial. The changes at the social level might not result in immediate measureable health benefits, but it is possible that this social change at the workplace could have an effect in the longer term. This would depend on whether the achieved changes can be sustained.

### Limitations of the Study

The time limit of this study prevented us from investigating long-term sustainability and effects of the social change, and a follow-up period of 6 months or a year would have allowed for an evaluation of long-term results. Furthermore, the dynamics in the different teams at different locations had a great impact on how the change came about. Further examination into these differences could help determine the factors that influence whether a tool has success or not. We only visited 4 out of 12 locations, and we cannot know whether other changes happened at the other locations. The fact that 90% of the study population was women also has to be considered when generalizing results into other contexts. Another weakness of this study is the lack of data on the individual reasons for dropout.

### Conclusions

Although having only a modest impact on individual participants’ lifestyle, a digital tool that encourages employees to participate in social activities at the workplace appears to initiate a social change in social and health care workers’ worksites, stimulating more social interaction around healthy lifestyle issues and habits. Future and more long-term studies are needed to decide whether such a change leads to sustained improvements in health. The potential role of social changes should be taken into consideration when designing and evaluating health promotion interventions. With regard to the BCT taxonomy of health promotion interventions, this study indicates that the category of social level behavior change techniques could be refined and described in more detail in the literature.

## Abbreviations

BCTs: behavior change techniques
SMS: short message service
